# Changes in Short-Term and Ultra-Short Term Heart Rate, Respiratory Rate, and Time-Domain Heart Rate Variability Parameters during Sympathetic Nervous System Activity Stimulation in Elite Modern Pentathlonists—A Pilot Study

**DOI:** 10.3390/diagnostics10121104

**Published:** 2020-12-17

**Authors:** Jakub S. Gąsior, Bartosz Hoffmann, Luiz Eduardo Virgilio Silva, Łukasz Małek, Andrew A. Flatt, Rafał Baranowski, Bożena Werner

**Affiliations:** 1 Department of Pediatric Cardiology and General Pediatrics, Medical University of Warsaw, 02-091 Warsaw, Poland; bozena.werner@wum.edu.pl; 2 Physiotherapy Division, Faculty of Medical Sciences, Medical University of Warsaw, 02-091 Warsaw, Poland; bartosz.hoffmann@icloud.com; 3 Department of Internal Medicine of Ribeirão Preto Medical School, University of São Paulo, Ribeirão Preto 14049-900, SP, Brazil; luizeduardo@usp.br; 4 Department of Epidemiology, Cardiovascular Disease Prevention and Health Promotion, National Institute of Cardiology, 04-635 Warsaw, Poland; lmalek@ikard.pl; 5 Biodynamics and Human Performance Center, Department of Health Sciences and Kinesiology, Georgia Southern University (Armstrong Campus), Savannah, GA 31419, USA; aflatt@georgiasouthern.edu; 6 Department of Heart Rhythm Disorders, National Institute of Cardiology, 04-628 Warsaw, Poland; rb@ikard.pl

**Keywords:** heart rate variability, heart rate, respiratory rate, modern pentathlon, athletes, physiological state, autonomic nervous system

## Abstract

Monitoring of markers reflecting cardiac autonomic activity before and during stressful situations may be useful for identifying the physiological state of an athlete and may have medical or performance implications. The study aimed to determine group and individual changes in short-term (5 min) and ultra-short-term (1 min) heart rate (HR), respiratory rate (RespRate), and time-domain heart rate variability (HRV) parameters during sympathetic nervous system activity (SNSa) stimulation among professional endurance athletes. Electrocardiographic recordings were performed in stable measurement conditions (Baseline) and during SNSa stimulation via isometric handgrip in 12 elite modern pentathlonists. Significant increases in short-term HR and decreases in time-domain HRV parameters with no changes in RespRate were observed during SNSa stimulation. Significant differences were observed between Baseline (all minutes) and the last (i.e., 5th) minute of SNSa stimulation for ultra-short-term parameters. Analysis of intra-individual changes revealed some heterogeneity in responses. The study provides baseline responses of HR, RespRate, and time-domain HRV parameters to SNSa stimulation among elite pentathlonists, which may be useful for identifying abnormal responses among fatigued or injured (e.g., concussed) athletes. More attention to individual analysis seems to be necessary when assessing physiological responses to sympathetic stimuli in professional endurance athletes.

## 1. Introduction

Elite athletes’ training process demands regular monitoring of fatigue and training response to optimize its effects and avoid over-training [1,2,3]. Autonomic regulation of the cardiocirculatory system is an important determinant of training adaptation [4,5]. Heart rate (HR) and heart rate variability (HRV) parameters are becoming increasingly popular as non-invasive and inexpensive biomarkers reflecting changes in parasympathetic and sympathetic activity [2,6,7]. Low resting HR and high HRV are beneficial to the performance of the sport [8,9]. It was shown that athletes who practice sports with attention tasks (e.g., pistol shooting, archery) and who presented fewer changes in stress related HRV measures performed better through improved accuracy [10,11]. Therefore, monitoring of parameters reflecting cardiac autonomic activity during the training session, shortly before and/or during professional competition may be useful in identifying the physiological state of an athlete [11,12], which can be further used to improve performance and consequently achieve better sport results. 

HRV alterations have been commonly analyzed on the basis of group changes in endurance athletes [13,14,15], which is ineffective in detecting individual athletes’ responses [16]. By analyzing individual HRV changes, one can identify athletes who show large or small responsiveness to the different stressors or challenges [16,17]. Consequently, it was suggested that HRV be assessed in athletes on an individual basis [4].

The isometric handgrip strength test is a non-invasive and validated tool used to stimulate cardiovascular and autonomic function [18,19,20]. Increased HR during isometric exercise [21] is due to vagal withdrawal [6,18,22,23] concurrent with increased sympathetic activity [6]. Clinicians have analyzed cardiovascular and/or hemodynamic responses to isometric exercise in different groups of patients, mostly using protocols with 2–6 min of sustained handgrip strength exercise [24,25,26,27,28,29]. There is a lack of studies assessing autonomic response to handgrip isometric exercise in athletes and how the HRV alterations to handgrip exercise can be used to assess the sport performance. Very recently, autonomic responsiveness to isometric handgrip has been investigated as a potential indicator of training fatigue [30]. Further investigation into practical assessment methodologies may influence protocols for monitoring athletic training status and wellbeing.

Coaches and sport practitioners strive for simplification of cardiovascular data acquisition in elite athletes [2,31,32]. Special attention has been given to (i) limiting the time needed to obtain reliable physiological outcomes and (ii) looking for parameters that can be used in the applied sports field. Several authors have demonstrated that vagally-mediated HRV parameters acquired from ultra-short-term 1 min electrocardiography recordings in elite athletes are reliable [31,32,33,34]. Recently, it was shown that in addition to commonly used HRV parameters, HR and respiratory rate calculated based on ultra-short-term 1 min recordings could be reliably used in elite endurance athletes [35]. 

The purpose of this study was to determine group and individual changes during sympathetic nervous system activity stimulation in short-term and ultra-short-term HR, respiratory rate, and HRV parameters commonly used in applied sports settings among elite modern pentathlonists. Determining the sensitivity of these metrics to the isometric stimulus may aid sports practitioners with HRV assessment during similar tasks performed in training and competition. 

## 2. Materials and Methods 

Details of the study participants and methods have been presented elsewhere [35]. A total of 12 elite modern pentathlonists (8♂) with professional careers ranging from 7 to 15 years, aged 17–26, participated in the study. Briefly, to be included in the study, participants should be an active athlete [36] currently in possession of the modern pentathlon license from the National Association; be in the pre-season period; declare the absence of diseases and/or regular use of medications affecting the cardiopulmonary system and/or interfering with the autonomic nervous system (ANS); accept and follow the measurement rules and protocol. To collect and control potential confounding variables influencing HRV, a questionnaire proposed by Laborde et al. [37] was used. The athletes were instructed to maintain normal sleep behaviors (as usual in the 5 days before examination), refrain from physical activity the day before and on the day of study, eat a normal, usual light breakfast, and use the toilet (if needed) on the day of study before examinations. The examinations were carried out at least 1 h after home breakfast and before lunch.

The study was approved by the University Ethical Committee and followed the rules and principles of the Helsinki Declaration (SKE 01-01/2017, 7 March 2017, Warsaw, Poland). All athletes gave their informed written consent. 

### 2.1. Electrocardiography Acquisition

Electrocardiography recordings (ECGs) were performed in a quiet, bright university room, with stable temperature and humidity. On the day of the study, each athlete underwent two ECG examinations. The first examination (6 min) was performed under established, controlled measurement conditions and was denoted as “Baseline” (B). The second examination (6 min) was performed during the sympathetic nervous system activity (SNSa) stimulation and was denoted as “SNSa stimulation” (SNSa). When the Baseline examination was finished, the athletes were instructed to grip the dynamometer, and SNSa stimulation examination was started. Twelve-lead ECG recordings (ECGs) (sampling frequency = 1000 Hz) were performed between 8:00 and 12:00 before lunch in a supine position using a portable personal computer with an integrated software system (Custo cardio 100 12-channel PC ECG system; Custo med GmbH, Ottobrunn, Germany). To stabilize HR and respiratory rate before starting appropriate ECG recordings (used to obtain RR intervals), athletes were instructed to lie in a supine position for 10 min [38] before Baseline recordings, and then the appropriate ECGs started. Athletes were encouraged to refrain from speaking and moving during the ECG examination. 

### 2.2. Sympathetic Nervous System Activity Stimulation

To stimulate SNSa, subjects were asked to grip the dynamometer (Saehan hydraulic hand dynamometer, model SH5001, Saehan Corporation, Masan, South Korea, second handle position) at 30% of their maximal voluntary contraction (MVC) using their dominant hand for a 6 min period. The 30% of MVC was controlled by one researcher (B.H.), and the athletes were informed and encouraged to squeeze continuously at 30%, maintaining the adequate value of MVC. To establish the 30% of MVC, a handgrip strength test was performed according to the guideline commonly used in adults [39] seven days before the study. 

### 2.3. Respiratory Rate

Respiratory rate (RespRate) during Baseline and SNSa stimulation was monitored using the Sony^®^ HDRAS20 Action Camera with Wi-Fi. The abdomen, thorax, and neck were recorded. The athletes were not instructed how to breathe but were informed that the breathing pattern would be video-recoded. Calculation of the RespRate based on 5 min and 1 min video recordings was independently performed by two researchers (B.H., J.S.G.).

### 2.4. HRV Analysis

The detailed ECGs data acquisition and processing have been described in our previous study [35]. Briefly, short-term and ultra-short-term HR and HRV parameters were calculated based on 5 min and 1 min segments, respectively, of appropriate ECGs (recordings started after stabilization period) using Kubios HRV Standard 3.4 software (University of Eastern Finland, Kuopio, Finland) [40,41]. The following parameters were calculated: heart rate (HR), standard deviation of normal-to-normal RR intervals (SDNN), root mean square of successive differences between adjacent normal RR intervals (RMSSD), the log-transformed RMSSD (lnRMSSD), log transformation of the ratio between RMSSD (in ms) and mean RR interval (mRR, in ms), i.e., lnRMSSD/mRR. 

Stationarity of the RR intervals and respiratory rate is required for the short-term frequency-domain HRV analysis [42,43]. The low- and high-frequency bands (LF and HF, respectively) of HRV are affected by (i) non-stationarities of the mean RR intervals and (ii) significant alterations or specified frequencies in breathing during recordings [37,44]. For instance, when any participant breathes very slowly, in the range of the LF band (~3 to 9 breaths per minute), the classical interpretation of the HF band as the vagal influence on the HR is flawed. Therefore, in studies where the immediate effect is measured, time-domain analysis is preferred. In the current study, HR increased, and RespRate decreased continuously during SNSa stimulation. Consequently, we assessed only time-domain HRV parameters. 

### 2.5. Relationships between Changes in HR and RespRate and Changes in HRV Parameters

The significant relationship between HRV parameters and mean HR has been usually overlooked in HRV studies [45,46,47]. HRV alterations should be interpreted by taking into account respective changes in resting HR [48]. In addition to HR, the correlation between differences in RespRate and differences in HRV parameters were also assessed in the present study.

### 2.6. Statistical Analysis

The Kolmogorov–Smirnov test was used to assess the normality of the data distribution. Natural log transformation (ln) was used if the data were not normally distributed. A paired Student’s *t*-test was employed to compare systematic changes between Baseline and SNSa stimulation in the analyzed parameters calculated based on 5 min recordings. One-way analysis of variance (ANOVA) with repeated measurements (with Tukey’s HSD post hoc test) was performed to compare the results of analyzed variables calculated based on 1 min recordings. To illustrate the relationship among differences between SNSa stimulation and Baseline in HRV parameters (Parameter-diff) and (a) participants characteristics (age, body mass index, career time) or (b) differences between SNSa stimulation and Baseline in HR (HR-diff) and RespRate (RespRate-diff) (Figure S1—Supplementary Materials), Pearson’s correlation coefficient (r) was calculated. The small sample size resulted in the figures with correlations being more useful as illustrative than as analytic. 

To detect individual athletes’ responses, tables with directional changes for all athletes individually are presented in Supplementary Materials (Tables S1 and S2). Recently, we showed acceptable reliability between Test and Retest of HR, RespRate, and HRV parameters calculated based on the first minute of the 5 min recordings [35]. In the current study, the parameters calculated based on the first minute of Baseline recordings were considered as a criterion for assessing parameters calculated based on the next minutes of the Baseline or 1st min of SNSa stimulation. The smallest worthwhile change (SWC) was calculated using formula 0.2 × standard deviation [49] of values from the 1st min of Baseline recordings (Criterion) to assess whether parameters: (a) calculated based on the next minutes of Baseline increased (↑) or decreased (↓) more than SWC or did not change (-) in comparison to Criterion; (b) calculated based on the 1st min of SNSa stimulation increased (↑) or decreased (↓) more than SWC or did not change (-) in comparison to the Baseline Criterion. Parameters calculated based on the next minutes of SNSa stimulation (from 2nd to 5th) were compared to the previous min of SNSa stimulation (Supplementary Materials, Tables S1 and S2). 

**Figure 1:**
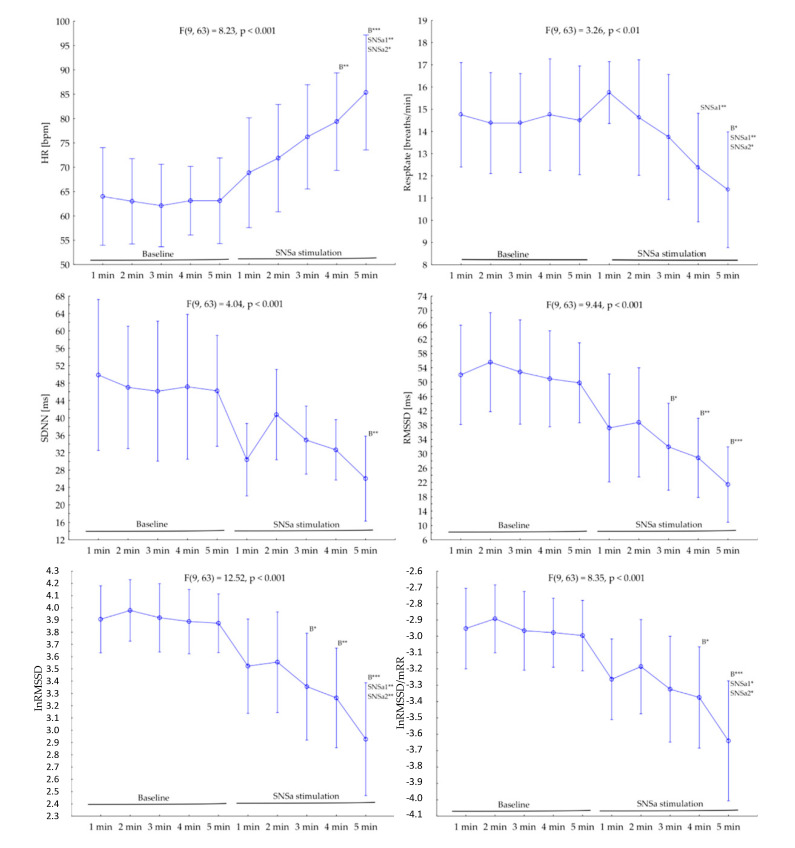
The group changes in heart rate (HR), respiratory rate (RespRate), and time-domain heart rate variability (HRV) parameters minute by minute during the Baseline and sympathetic nervous system activity (SNSa) stimulation recordings. B—significant difference versus Baseline (all minutes), SNSa1—significant difference versus the 1st min from SNSa stimulation, SNSa2—significant difference versus the 2nd min from SNSa stimulation, * *p* < 0.05, ** *p* < 0.01, *** *p* < 0.001.

**Figure 2:**
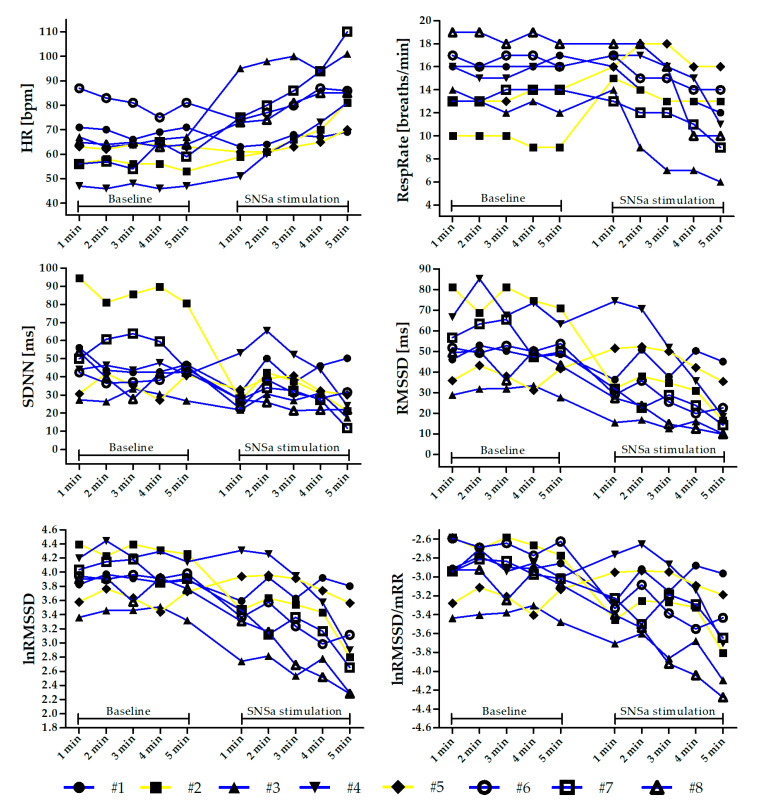
Individual changes in heart rate (HR), respiratory rate (RespRate), and heart rate variability (HRV) parameters minute by minute during the Baseline and sympathetic nervous system activity (SNSa) stimulation recordings. Individuals highlighted in yellow (#2 and #5) are those who increased the RespRate during SNSa stimulation.

**Figure 3:**
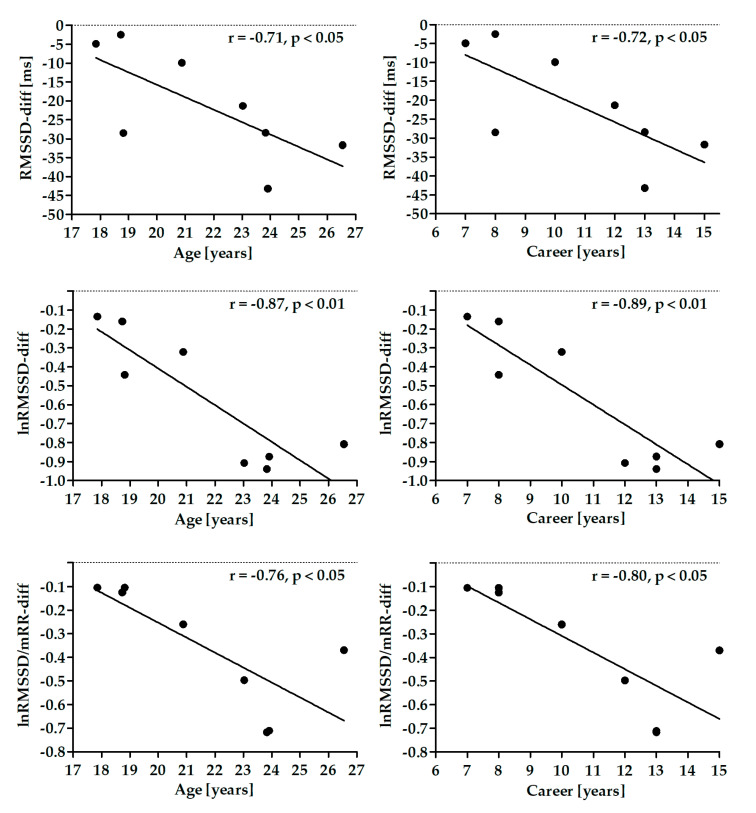
Correlation between differences in root mean square of successive differences (RMSSD) parameters and athlete’s age and career time.

The threshold probability of *p* < 0.05 was taken as the level of significance for all statistical tests. Figure 1 was created and all calculations were performed using STATISTICA 12 (StatSoft Inc., Tulsa, OK, USA). GraphPad Prism 5 (GraphPad Software Inc., San Diego, CA, USA, 2005) was used to create Figure 2 and Figure 3 and Figure S1.

## 3. Results

### 3.1. Participants Information

Results of 8 male athletes were included in the statistical analysis (results of 4 participants out of 12 were excluded due to the detection of prolonged QTc interval > 450 ms, n = 2; left bundle branch block, n = 2). The mean (±SD) age, weight, height, body mass index (BMI), and duration of professional athletic career were: 21.7 years (±3.1), 75.9 kg (±9.5), 182.6 cm (±6.1), 22.7 kg/m^2^ (±2.3), and 10.8 years (±2.9). Athletes declared participating in 19 training sessions (±2) per week during the normal in-season time.

### 3.2. Changes in Short-Term 5 min Parameters

Table 1 presents the results of short-term 5 min HR, RespRate, and HRV parameters from Baseline and SNSa stimulation. There was a significant increase in HR and a significant decrease in SDNN, RMSSD, lnRMSSD, and lnRMSSD/mRR. No significant changes were observed for RespRate.

### 3.3. Changes in Ultra-Short Term 1-min Parameters

A one-way repeated-measures ANOVA revealed that there was a significant main effect of SNSa stimulation in all analyzed parameters (F between 3.26 and 12.52). Post hoc tests showed that there were no significant differences between 1 min parameters from 5 min ECG Baseline recordings in all analyzed indices (Figure 1). However, analysis of intra-individual changes revealed that some athletes presented worthwhile alterations between parameters calculated based on the 1st min (named Criterion) and the next minutes during Baseline recordings (Figure 2 and Tables S1 and S2). 

HR gradually increased during SNSa stimulation. HR_(SNSa4)_ (mean: 79.4 ± 12 bpm) and HR_(SNSa5)_ (85.4 ± 14.1 bpm) were significantly higher than HR_(B)_ (all minutes—between 62.1 and 64.0 bpm), and HR_(SNSa5)_ was significantly higher than HR_(SNSa1)_ (68.9 ± 13.5 bpm) and HR_(SNSa2)_ (71.9 ± 13.2 bpm). Interestingly, two athletes (#1 and #6) presented a worthwhile decrease in HR values during the 1st min of SNSa stimulation in comparison to the Criterion from Baseline. A similar pattern of changes during the next minute of SNSa stimulation, i.e., increasing HR values, was observed for most athletes. 

RespRate initially increased during SNSa stimulation and then gradually decreased with time. RespRate from the last minute of SNSa stimulation (RespRate_(SNSa5)_ 11 ± 3 breaths/min) was significantly lower than RespRate from Baseline (RespRate_(B)_ all minutes—between 14 and 15 breaths/min), 1st min (RespRate_(SNSa1)_ 16 ± 2 breaths/min) and 2nd min (RespRate_(SNSa2)_ 15 ± 3 breaths/min) of SNSa stimulation. Not all athletes presented the same pattern of breathing rate alterations during SNSa stimulation. Two athletes increased and then slightly decreased the RespRate (#2 and #5—yellow lines), whereas others monotonically decreased the RespRate (blue lines, Figure 2).

Generally, a similar pattern of changes during SNSa stimulation was observed for all time-domain HRV parameters. The first minute of SNSa stimulation was accompanied by a nominal decrease in SDNN, RMSSD, lnRMSSD, and lnRMSSD/mRR compared to Baseline. Then, a nominal increase followed by a gradual decrease in all parameters was observed. 

Statistically significant differences were observed between Baseline (all minutes) and last minute (5th) of SNSa stimulation (Figure 1). Detailed results are presented as follows: SDNN: _(B)_ between 46.1 ms and 49.9 ms vs. _(SNSa5)_ 26.1 ± 11.7 ms; RMSSD: _(B)_ between 49.8 ms and 55.6 ms vs. _(SNSa5)_ 21.4 ± 12.5 ms; lnRMSSD: _(B)_ between 3.87 and 3.98 vs. _(SNSa5)_ 2.93 ± 0.55; lnRMSSD/mRR: _(B)_ between −2.89 and −3.00 vs. _(SNSa5)_ −3.64 ± 0.44. 

Additionally, lnRMSSD_SNSa5_ and lnRMSSD/mRR_SNSa5_ presented significantly lower values than these parameters calculated based on the 1st min of SNSa stimulation (lnRMSSD_SNSa1_: 3.52 ± 0.46 and lnRMSSD/mRR_SNSa1_: −3.26 ± 0.29) and the 2nd min of SNSa stimulation (lnRMSSD_SNSa2_: 3.55 ± 0.49 and lnRMSSD/mRR_SNSa2_: −3.19 ± 0.35). 

A nominal decrease in vagally-mediated time-domain HRV parameters (RMSSD, lnRMSSD) during the 1st min of SNSa stimulation, observed for the group statistic, was not presented by all athletes when assessed individually. Worthwhile increases in these parameters in comparison to Baseline Criterion were noted for two athletes (#4 and #5). 

### 3.4. Relationship among Differences between SNSa Stimulation and Baseline in HRV Parameters and Participants’ Characteristics 

There were no significant correlations between age, BMI and career time and differences in HR, RespRate, and SDNN. Differences in RMSSD parameters were significantly negatively correlated with age and career time (Figure 3). There were no significant correlations between these parameters and BMI. 

### 3.5. Correlation between Differences in HRV Parameters and Differences in HR or RespRate

The correlations for SNSa–Baseline differences between HRV parameters and HR (column A) or RespRate (column B) are shown as Supplementary Materials in Figure S1. For 5 min short-term RMSSD, lnRMSSD, and lnRMSSD/mRR, SNSa stimulation–Baseline differences are more correlated to the differences in HR (HR-diff) than to the differences in RespRate (RespRate-diff). For short-term SDNN, SNSa stimulation–Baseline difference was more correlated with the RespRate-diff than the HR-diff. Significant, negative correlations were observed for the association between HR-diff and lnRMSSD-diff (r = −0.72, *p* < 0.05).

## 4. Discussion

In the current study, we explored grouped and individual responses of short-term and ultra-short-term HR, RespRate, and time-domain HRV parameters to sympathetic nervous system activity stimulation in elite modern pentathlonists. When analyzing the whole group, we observed a significant increase in short-term (5 min) HR and a significant decrease in short-term SDNN and vagally-mediated HRV parameters. There was no significant group change in RespRate, even though the mean values decreased nominally. 

Sustained isometric exercise using a handgrip dynamometer has been commonly used by clinicians to compare cardiovascular and hemodynamic responses between groups of patients and healthy controls [27,29,50,51,52]. In athletes, isometric handgrip contraction has been used less frequently to evaluate cardiac autonomic modulation during physical exertion [53]. Abaji et al. compared HRV results between groups of concussed and control athletes [53]. Authors observed significantly different responses between groups—post-concussion athletes showed significantly lower vagally-mediated high-frequency power calculated based on the 3 min recordings during isometric handgrip contraction (30% MVC). They concluded that monitoring of HRV in athletes may aid diagnosis and provide insight into the safe return to play [53]. Very recently (2020), a research group in Spain published a protocol with the objective to determine changes in the performance of professional athletes following an HRV-guided training period [3]. By referencing studies published in recent years [54,55,56], authors stated that HRV-guided training in endurance athletes enables sports practitioners to better adapt training loads to an individual athlete in search of a better recovery and sports outcomes [3]. 

No significant differences were observed between each 1 min segment and the Criterion 1st min from 5 min ECG Baseline recordings in all analyzed indices, in line with a study by Flatt and Esco from 2016 [31]. Authors also demonstrated that randomly selected 1 min lnRMSSD segments within a standard 5-min ECG recording period were no different from the Criterion among collegiate athletes [32]. 

In our opinion, the main observation could be focused on parameters calculated based on the last minute from Baseline recordings and the first min of SNSa stimulation recordings. The isometric handgrip exercise is not a sports competition. Nevertheless, the effect and procedure of squeezing the dynamometer’s handle may be, to some extent, considered as pre-competition stress and comparable to, e.g., shooting performance. We did not observe statistically significant differences in all measured indices between parameters calculated based on the last min (5th) of the Baseline recordings and the first min of SNSa stimulation recordings. However, the lack of statistical significance does not imply that the intervention/stimulus was not clinically or practically relevant [57]. In the current study, analysis of intra-individual alterations in analyzed parameters during SNSa stimulation revealed different worthwhile athletes’ responses to the applied stimulus. In the whole group, HR and RespRate increased, and HRV parameters decreased during 1st min of SNSa stimulation. However, not all of the elite endurance athletes presented the same pattern of alterations. For example, two athletes (#2 and #5) started to breathe faster during SNSa stimulation, and their HRs increased only slightly. However, for athlete #2, a decrease and for athlete #5, an increase in vagally-mediated HRV parameters was observed, respectively. Therefore, our findings support the suggestion that HRV must be assessed in athletes on an individual basis [4,16]. 

Fewer changes in stress related HRV parameters during attention tasks, such as pistol shooting, improved accuracy [10,11]. Interestingly, changes resulting from SNSa stimulation in short-term (5 min) vagally-mediated HRV parameters were higher in athletes with age and longer careers. A long-term professional career is associated with electrocardiographic alterations in endurance elite athletes [58]. This group has an elevated parasympathetic activity, consequently lower HR and higher baseline HRV compared to, e.g., recreational athletes or non-athletic matched controls [58]. High baseline HRV has a greater margin to decrease. Inversely, low HRV cannot decrease too much lower, especially with concomitant high values of HR. Taylor et al. underlined that a smaller tachycardiac response to isometric exercise in non-athlete older compared to younger men is associated with an inability to decrease cardiac vagal tone below an already reduced baseline level [18]. In our study, there was no significant correlation between baseline HR or HRV and professional career, i.e., athletes with longer professional career did not present lower HR and/or higher HRV than athletes with shorter careers. Therefore, higher changes in short-term vagally-mediated HRV parameters resulted from SNSa stimulation in athletes with longer careers were not related to high baseline values in these groups of athletes.

Not unexpectedly, alterations resulting from SNSa stimulation in lnRMSSD were associated with the changes in HR. A previous study showed that changes in time-domain HRV parameter values obtained from two measurements performed during stable conditions with a one-week interval between tests without external stimulus were shown to be affected by differences in HR in this group of athletes [35]. Even a minimal change in HR considerably altered HRV [59]. Consequently, changes in HRV parameters in sports practitioners should be analyzed, taking into account concomitant changes in HR, such as lnRMSSD/mRR [2,35]. Here, lnRMSSD/mRR also showed differences between Baseline and SNSa stimulation, demonstrating that the vagal modulation is altered during the handgrip test and is not a simple consequence of HR changes.

To check if the autonomic responsiveness, i.e., the HRV differences between SNSa stimulation and Baseline, is related to the athlete’s characteristics (age, BMI, and career time), the correlation was analyzed. RMSSD parameters were negatively correlated to the athlete’s age and career time. However, since correlation plots are very similar for the two characteristics, it is very likely that the correlation between RMSSD and career time is dependent on the athlete’s age. It is known that RMSSD decreases with the natural aging process [60]. Therefore, we believe that the different age is, at least in part, responsible for the individual differences in the autonomic responsiveness to the handgrip test. On the other hand, it does not explain why some individuals increased RespRate during SNSa stimulation while others did not.

Uncontrolled variables within experimental conditions may significantly influence HRV results [61]. Careful consideration of such crucial contextual, environmental, physiological, and methodological factors is required to obtain more accurate and reproducible results. Time of day to record the data (in the case of short-time recordings); subject-characteristic variables (health and physical activity status, control for medication, food and water consumption, voiding of the bladder); the position of the body during short-time recordings; the quality of recorded signals (recording period length, detection or recording method, sampling frequency, breathing pacing—paced or free breathing); as well as the tools used to analyze HRV values (software, removal of artifacts) are examples of such factors [61,62,63,64]. In the current study, to collect and control most of the mentioned variables influencing HRV, we used the questionnaire proposed by Laborde et al. [37].

Limitations of the current study were pointed out previously [35]. The small sample of only males and cross-sectional study design limits the generalizability of the study’s findings and hinders sex subgroup comparisons. HRV data were obtained during supine ECG recordings performed in controlled laboratory settings. HR monitors may be more practical to collect RR intervals in athletes than traditional ECG, especially during sports competition [65]. However, RR intervals obtained using an HR monitor may add uncertainty to the analysis and interpretation of HRV [66,67]. ECG screening among professional athletes before including the data for detailed HRV analysis was suggested [35]. 

Electrocardiographic pre-participation screening followed by periodic ECG monitoring in competitive athletes has been established as an easily accessible and effective first-line test in diagnostics of various cardiovascular disorders, which may put athletes at risk of potentially life-threatening events [68]. ECG can be used to detect the signs of a physiological adaptation to exercise called athlete’s heart and to differentiate them from potentially pathologic changes, which should require further management [69]. Electrocardiographic monitoring during exercise can be used to assure a linear increase in HR in parallel with increasing exercise intensity until the individual’s maximal HR and to analyze HR recovery post-exercise. These data can then be utilized to optimize the training effects or to detect early signs of overtraining characterized, among others, by decreased maximal HR and prolonged HR recovery [70]. Finally, both overtraining and pathological heart conditions may manifest as supraventricular and ventricular arrhythmias during exercise or at rest detected by various ECG monitoring tools, which have been continuously adapted to the sporting environment [71]. 

In the current study, we did not evaluate cardiac function using echocardiography. Echocardiography is currently not a part of the routine pre-participation assessment of athletes [72]. It is usually performed in cases of suspected abnormalities after personal and family history, physical examination, or ECG. However, it may potentially disclose changes undetected by routine screening, including valvular disease (bicuspid aortic valve, mitral valve prolapse), coronary artery anomalies in younger athletes and dilatation of the aorta, late-onset cardiomyopathies, and wall motion abnormalities due to myocarditis or coronary artery disease in older athletes [73]. For these reasons, there is a growing belief that if it is available and reliable, it may be added to the baseline pre-participation screening panel in athletes.

## 5. Conclusions

Our findings provide baseline responses of short-term and ultra-short-term HR, RespRate, and time-domain HRV parameters to sympathetic nervous system activity stimulation among elite modern pentathletes that can be used in future studies for comparison with, e.g., concussed pentathletes. These data show “normal” responses, which may, therefore, aid in the identification of abnormal responses (as well as recovery) among concussed athletes. Analysis of inter-individual responses of modifiable parameters (e.g., breathing rate) to a specified stimulus may help in the identification of athletes that will benefit from practical techniques aimed at avoiding pre-performance stress, improve performance in sports and achieve better sport results. The handgrip test can be used as a tool in the analysis of autonomic responsiveness to sympathetic stimulation in pentathletes, with potential application to athletes from other modalities of sports.

## Figures and Tables

**Table 1:** The effect of sympathetic nervous system activity (SNSa) stimulation on short-term 5 min heart rate (HR), respiratory rate (RespRate), and time-domain heart rate variability (HRV) parameters.

	Baseline	SNSa Stimulation	*p*
HR [bpm]	62.9 ± 9.7	77.8 ± 12.1	<0.05
RespRate [breaths per min]	15 ± 3	13 ± 3	0.41
SDNN [ms]	45.9 ± 20.0	26.8 ± 6.0	<0.05
RMSSD [ms]	48.2 ± 21.2	26.9 ± 12.2	<0.01
lnRMSSD	3.78 ± 0.49	3.21 ± 0.44	<0.01
lnRMSSD/mRR	−3.09 ± 0.35	−3.45 ± 0.34	<0.01

SNSa—sympathetic nervous system activation; HR—heart rate; RespRate—respiratory rate; SDNN—standard deviation of normal-to-normal RR intervals; RMSSD—root mean square of successive differences between adjacent normal RR intervals; mRR—mean RR interval; ln—log-transformed.

## Supplementary Materials

The following are available online at https://www.mdpi.com/2075-4418/10/12/1104/s1, Figure S1: Relationship among differences between sympathetic nervous system activity (SNSa) stimulation and Baseline in heart rate variability (HRV) parameters (Parameter-diff) and heart rate (HR) (HR-diff) (A) and respiratory rate (RespRate) (RespRate-diff) (B), Table S1: Individual athletes’ responses in heart rate (HR) and respiratory rate (RespRate) during sympathetic nervous system activity (SNSa) stimulation, Table S2: Individual athletes’ responses in standard deviation of normal-to-normal RR intervals (SDNN) and root mean square of successive differences (RMSSD) parameters during sympathetic nervous system activity (SNSa) stimulation.
