# Supplementary material for: Changes in Short-Term and Ultra-Short Term Heart Rate, Respiratory Rate, and Time-Domain Heart Rate Variability Parameters during Sympathetic Nervous System Activity Stimulation in Elite Modern Pentathlonists—A Pilot Study
Source: Diagnostics (Basel). 2020 Dec 17;10(12):1104. doi: 10.3390/diagnostics10121104 (PMC7766436; doi:10.3390/diagnostics10121104)
Supplement: Supplementary file 1 [file diagnostics-10-01104-s001.pdf]

Table S1. Individual athletes' responses in heart rate (HR) and respiratory rate (RespRate) during sympathetic nervous system activity (SNSa) stimulation.

	SWC	#	**Baseline**					**SNSa stimulation**				
			1 min Criterion	2 min	3 min	4 min	5 min	1 min	2 min	3 min	4 min	5 min
**HR [bpm]**	2.4	1	71	-	↓	-	-	↓	-	↑	-	-
		2	56	-	-	-	↓	↑	-	↑	↑	↑
		3	67	↓	↓	-	-	↑	↑	-	↓	↑
		4	47	-	-	-	-	↑	↑	↑	↑	↑
		5	63	-	-	-	-	-	-	-	-	↑
		6	87	↓	↓	↓	↓	↓	↑	↑	↑	-
		7	56	-	-	↑	↑	↑	↑	↑	↑	↑
		8	65	-	-	-	-	↑	-	↑	↑	-
**RespRate [breaths/min]**	1	1	16	-	-	-	-	-	↓	-	-	-
		2	10	-	-	-	-	↑	-	-	-	-
		3	14	-	↓	-	↓	-	↓	↓	-	-
		4	16	-	-	-	-	-	-	-	-	↓
		5	13	-	-	-	-	↑	↑	-	↓	-
		6	17	-	-	-	-	-	↓	-	-	-
		7	13	-	-	-	-	-	-	-	-	↓
		8	19	-	-	-	-	-	-	↓	↓	-

↑ – increased / ↓ – decreased more that smallest worthwhile change (SWC) or not (-); Baseline: in comparison to 1 min Criterion; sympathetic nervous system activity (SNSa) stimulation: 1st min: ↑ – increased / ↓ – decreased more that SWC or not (-) in comparison to 1 min Criterion from Baseline; next minutes (2nd, 3rd, 4th, 5th) of SNSa stimulation: comparison to previous min of SNSa stimulation

Table S2. Individual athletes' responses in standard deviation of normal-to-normal RR intervals (SDNN) and root mean square of successive differences (RMSSD) parameters during sympathetic nervous system activity SNSa stimulation.

			**Baseline**					**SNSa stimulation**				
	SWC	#	1 min	2 min	3 min	4 min	5 min	1 min	2 min	3 min	4 min	5 min
**SDNN [ms]**	4.2	1	56.0	↓	↓	↓	↓	↓	↑	↓	↑	-
		2	94.6	↓	↓	↓	↓	↓	↑	↓	↓	↓
		3	27.3	-	↑	-	-	↓	↑	-	-	↓
		4	44.1	-	-	-	-	↑	↑	↓	↓	↓
		5	30.7	↑	↑	-	↑	-	↑	-	↓	-
		6	42.4	↓	↓	-	-	↓	↑	↓	-	-
		7	50.0	↑	↑	↑	↓	↓	↑	-	↓	↓
		8	53.8	↓	↓	↓	↓	↓	-	↓	-	-
**RMSSD [ms]**	3.3	1	46.0	↑	↑	-	-	↓	↑	↓	↑	↓
		2	81.2	↓	-	↓	↓	↓	↑	-	↓	↓
		3	28.8	-	-	↑	-	↓	-	↓	↑	↓
		4	66.6	↑	-	↑	↓	↑	↓	↓	↓	↓
		5	35.8	↑	-	↓	↑	↑	-	-	↓	↓
		6	51.7	-	-	-	-	↓	↑	↓	↓	-
		7	56.6	↑	↑	↓	↓	↓	↓	↑	↓	↓
		8	49.4	-	↓	-	↓	↓	↓	↓	-	↓
**lnRMSSD**	0.07	1	3.83	↑	↑	-	-	↓	↑	↓	↑	↓
		2	4.40	↓	-	↓	↓	↓	↑	↓	↓	↓
		3	3.36	↑	↑	↑	-	↓	-	↓	↑	↓
		4	4.20	↑	-	↑	-	↑	-	↓	↓	↓
		5	3.58	↑	-	↓	↑	↑	-	-	↓	↓
		6	3.95	-	-	-	-	↓	↑	↓	↓	↑
		7	4.04	↑	↑	↓	↓	↓	↓	↑	↓	↓
		8	3.90	-	↓	-	↓	↓	↓	↓	↓	↓
**lnRMSSD/mRR**	0.06	1	-2.91	↓	-	-	-	↑	↓	↑	↓	↑
		2	-2.58	↑	-	↑	↑	↑	↓	-	-	↑
		3	-3.44	-	-	↓	-	↑	↓	↑	↓	↑
		4	-2.95	↓	-	↓	-	↓	↓	↑	↑	↑
		5	-3.28	↓	↓	↑	↑	↓	-	-	↑	↑
		6	-2.59	↑	-	↑	-	↑	↓	↑	↑	↓
		7	-2.94	↓	↓	-	↑	↑	↑	↓	↑	↑
		8	-2.93	-	↑	-	↑	↑	↑	↑	↑	↑

↑ – increased / ↓ – decreased more that smallest worthwhile change (SWC) or not (-); Baseline: in comparison to 1 min Criterion; sympathetic nervous system activity (SNSa) stimulation: 1st min: ↑ – increased / ↓ – decreased more that SWC or not (-) in comparison to 1 min Criterion from Baseline; next minutes (2nd, 3rd, 4th, 5th) of SNSa stimulation: comparison to previous min of SNSa stimulation

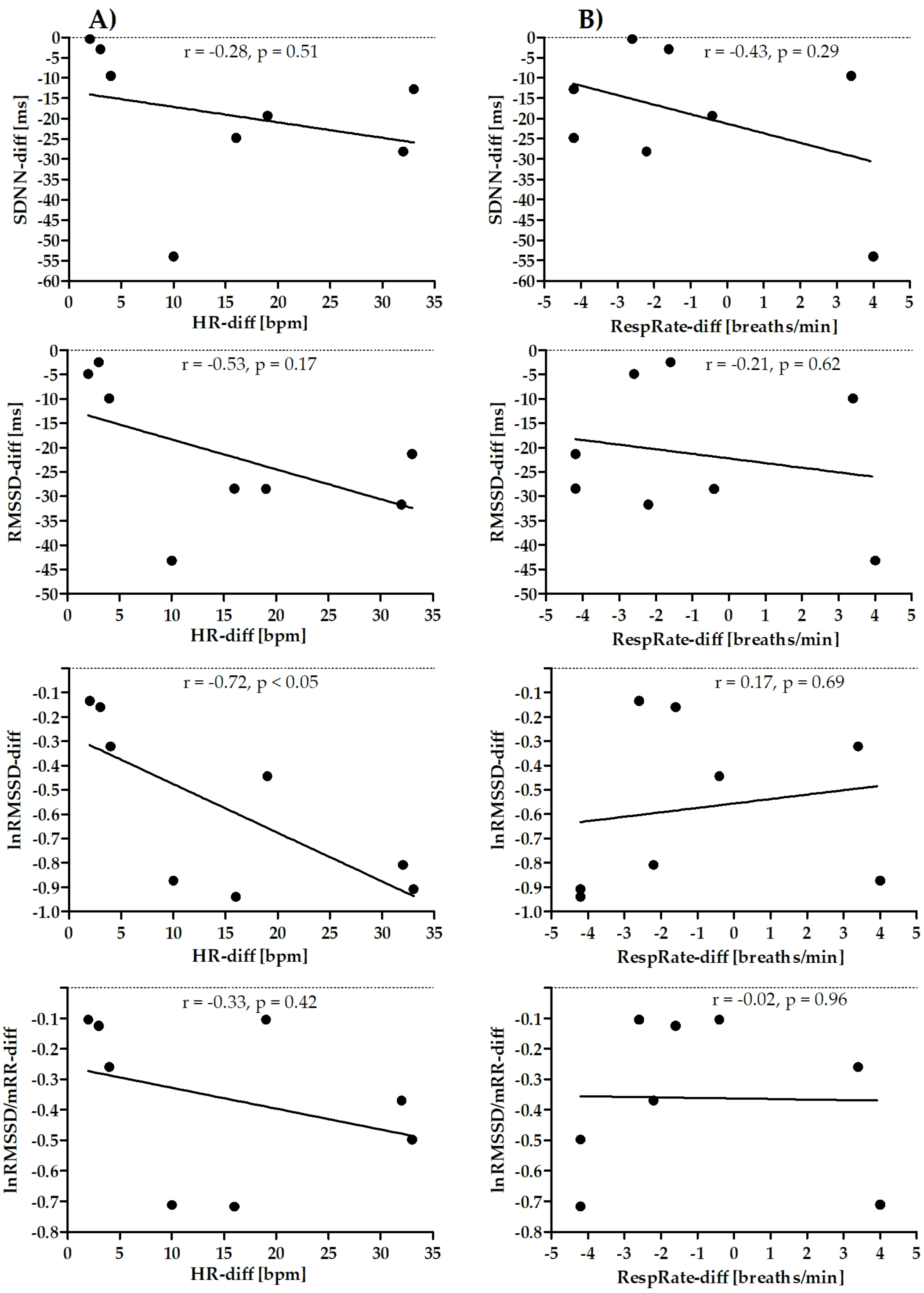

Figure S1. Relationship among differences between sympathetic nervous system activity (SNSa) stimulation and Baseline in heart rate variability (HRV) parameters (Parameter-diff) and heart rate (HR) (HR-diff) (A) and respiratory rate (RespRate) (RespRate-diff) (B).
